# Plant–soil feedbacks benefit plant invaders under multiple global change factors

**DOI:** 10.1093/nsr/nwag415

**Published:** 2026-07-08

**Authors:** Cai Cheng, Yanjie Liu, Jihua Wu, Mark van Kleunen

**Affiliations:** State Key Laboratory of Wetland Conservation and Restoration, National Observations and Research Station for Wetland Ecosystems of the Yangtze Estuary, Ministry of Education Key Laboratory for Biodiversity Science and Ecological Engineering and Institute of Eco-Chongming, School of Life Sciences, Fudan University, Shanghai 200438, China; Department of Biology, University of Konstanz, Konstanz 78464, Germany; State Key Laboratory of Black Soils Conservation and Utilization, Northeast Institute of Geography and Agroecology, Chinese Academy of Sciences, Changchun 130102, China; State Key Laboratory of Wetland Conservation and Restoration, National Observations and Research Station for Wetland Ecosystems of the Yangtze Estuary, Ministry of Education Key Laboratory for Biodiversity Science and Ecological Engineering and Institute of Eco-Chongming, School of Life Sciences, Fudan University, Shanghai 200438, China; State Key Laboratory of Herbage Improvement and Grassland Agro-Ecosystems, College of Ecology, Lanzhou University, Lanzhou 730000, China; Department of Biology, University of Konstanz, Konstanz 78464, Germany; Zhejiang Provincial Key Laboratory of Plant Evolutionary Ecology and Conservation, Taizhou University, Taizhou 318000, China; Zhejiang Key Laboratory for Restoration of Damaged Coastal Ecosystems, School of Life Sciences, Taizhou University, Taizhou 318000, China

**Keywords:** arbuscular mycorrhizal fungi, Anthropocene, exotic species, multiple factors, soil-legacy effects

## Abstract

Plant–soil feedbacks (PSFs) are an important mechanism determining plant community dynamics and invasion by non-native species. While the individual responses of plants and soil microbes to multiple concurrent global change factors (GCFs) have been documented, it remains poorly understood how multiple GCFs affect PSFs and, particularly, whether this effect depends on plant origin. We conducted a PSF experiment with five pairs of native and alien species and nine GCFs applied in concurrent combinations of 0, 1, 3 and 6 GCFs. As GCF number increased, PSFs shifted from negative to neutral or positive in native species, but were consistently positive and became stronger in alien species. The positive response of PSFs to multiple GCFs was due to specific interactions between coacting GCFs in native species, but not in alien species, and was related to changes in soil mutualists. Our study highlights that PSFs could promote the spread of alien species under multiple concurrent GCFs.

## INTRODUCTION

Anthropogenic activities are shaping ecosystems worldwide in unprecedented ways, triggering a variety of environmental changes like warming, drought, nitrogen deposition, soil salinization, microplastic pollution and the use of various biocides [1–3]. Although the individual consequences of many of these global change factors (GCFs) have been well documented, a growing body of studies highlights that ecosystems are simultaneously exposed to multiple GCFs with different physical, chemical and biological attributes, which may have unpredictable consequences due to synergistic or antagonistic interactions [4–6]. For example, Rillig *et al.* [7] reported that increasing the number of simultaneously acting GCFs exacerbated the negative impacts on soil respiration and fungal diversity. The ecological effects of multiple GCFs have also been tested on plants, with significant negative impacts on plant growth [8] and community diversity [9]. While these studies provide important insights into the separate responses of plants and soil biota to multiple coacting GCFs, few have taken the next step to test the response of plant–soil interactions [10].

Plant–soil feedbacks (PSFs) have important implications for plant species coexistence, community dynamics and ecosystem processes and functioning [11–14]. PSF refers to the process in which plants alter biotic and abiotic soil properties, which in turn either promote or suppress the performance of subsequent plants [15,16]. Negative conspecific PSFs occur when plants grow worse on soil that has been conditioned by conspecifics compared to control soil (e.g. sterilized, unconditioned or conditioned by other species) [17], often as a result of pathogen accumulation, nutrient depletion or loss of mutualists [18]. Conversely, if mutualist accumulation and/or a loss of pathogen pressure leads to better growth on conspecific soil than on control soil, positive PSFs can emerge and enable the plant species to become dominant [18]. When comparing plant growth on conspecific soil to growth on unconditioned soil, PSFs capture the response of plants to total changes in biotic and abiotic soil properties (i.e. total PSFs) [17]. Conversely, plants are also frequently tested on soil conditioned by other species (heterospecifics) and compared to growth on conspecific soil, thereby evaluating the response of plants to species-specific changes in biotic and abiotic soil properties (i.e. specific PSFs) [17].

Ample evidence indicates that PSFs are highly context-dependent and strongly modulated by the external environment [19–21]. For example, drought can weaken the strength of negative PSFs [22–24], likely due to reduced pathogen influences and/or amplified mutualist contributions to plant growth [25,26]. Conversely, negative PSFs can be intensified by eutrophication [27,28], possibly because plants become less dependent on soil mutualists and suffer from higher pathogen pressure in nutrient-rich environments [25,29]. Although multiple studies indicate the importance of single GCFs to the strength and direction of PSFs, the influence of multiple coacting GCFs on PSFs is much less well understood.

A recent study reports that increasing the number of GCFs during the testing phase altered soil fungal legacies left by the invasive alien species *Hydrocotyle verticillata*, resulting in positive total PSFs [30]. Whether this finding for a single specific species can be generalized to other species or to more realistic cases where multiple GCFs occurred in both the training and testing phases, however, is unclear. Indeed, whether plants are native to a given region or alien can strongly influence the nature and strength of PSFs [31–33]. In ambient environments, native species often experience negative PSFs, whereas aliens generally experience neutral or even positive PSFs by having escaped from soil-borne pathogens and/or by establishing stronger interactions with soil mutualists [34–37]. Nevertheless, whether PSFs respond differently to multiple coacting GCFs in alien versus native plant species remains unknown.

Here, we tested whether the number of GCFs affects PSFs, and whether this differs between native and alien plant species. We conducted a PSF pot experiment with five pairs of alien and native grassland species under diverse GCF treatments in an outdoor common garden (Fig. S1). In the training phase, we conditioned soil by each of the 10 species (conspecific soil), with unplanted soil as the control. To distinguish between specific PSFs and total PSFs, we also conditioned soil by a common native species (*Plantago lanceolata*) as a heterospecific control. In the testing phase, we planted the 10 species in the aforementioned three soil-conditioning treatments and used plant total biomass, the colonization by arbuscular mycorrhizal fungi (AMF) and nodule number to calculate PSFs. To test the response of PSFs to multiple coacting GCFs, we exposed both the training and testing phases to four levels of GCF numbers (0, 1, 3 and 6) based on a pool of nine common GCFs, namely, drought, nitrogen deposition, light pollution, fungicide use, insecticide use, antibiotics, soil salinization, microplastics of the synthetic rubber ethylene propylene diene monomer (EPDM) and car-tire microplastics (Fig. S1b). According to previous evidence that environmental conditions become more stressful with an increasing number of GCFs [7,8] and that mutualist benefits increase while pathogen pressure decreases in response to increasing stress intensity [25], we hypothesized that multiple coacting GCFs would promote positive PSFs, and that such effects would be stronger in native species than in alien species.

## RESULTS

### Responses of plant biomass to multiple GCFs in the training phase

**Figure 1.**
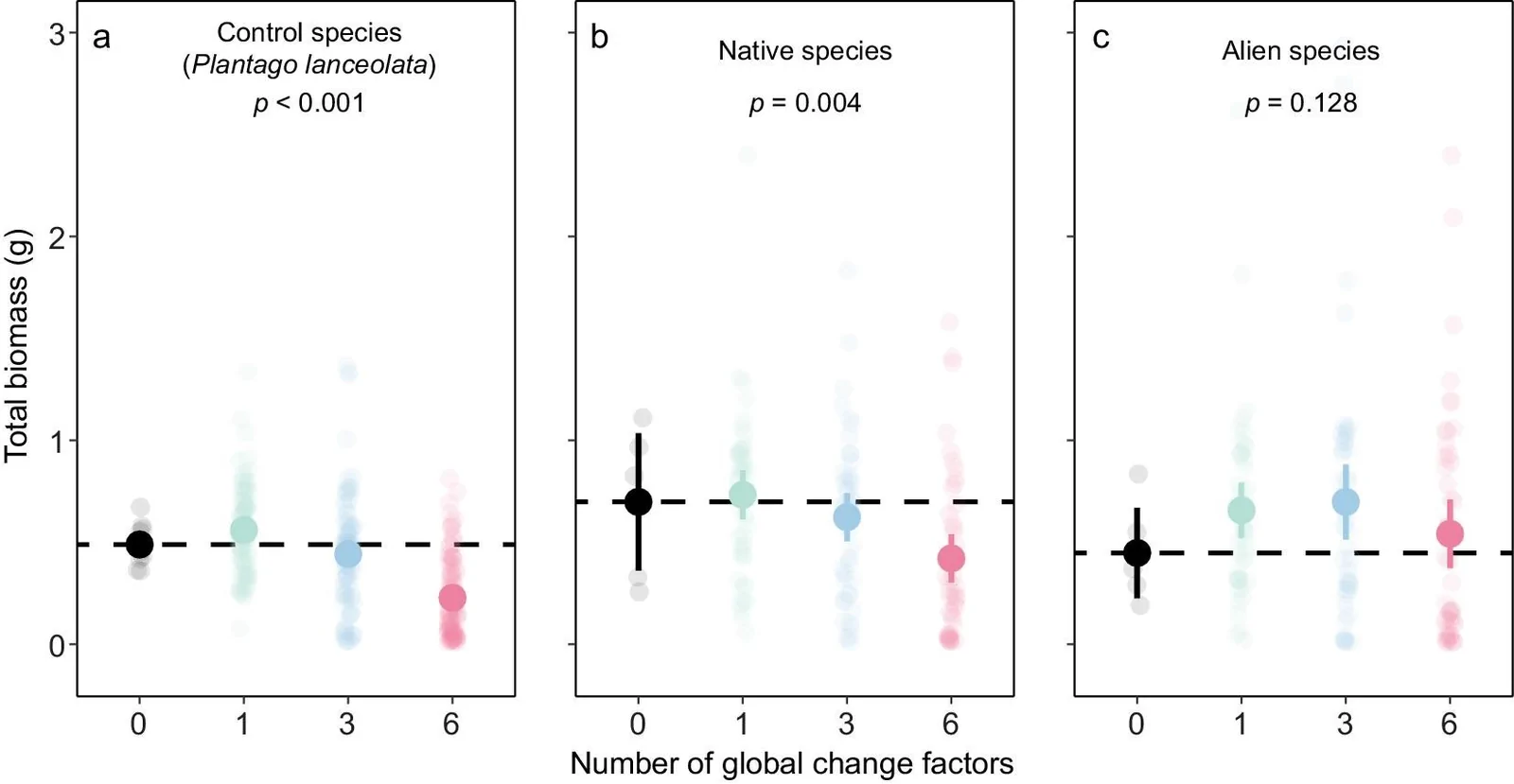
Total biomass of plants under different numbers of GCFs in the training phase. (a) The control species *P. lanceolata*, (b) the native species and (c) the alien species. Points with error bars are means and 95% confidence intervals. The black dashed line indicates the mean of plant biomass under the ambient condition without GCFs.

In the training phase, most of the single GCFs did not alter plant biomass, except for the positive effect of nitrogen deposition on the alien species and on the control species *P. lanceolata*, and the negative effect of soil salinization on *P. lanceolata* (Fig. S2). However, an increasing number of GCFs significantly decreased total biomass for the native species and *P. lanceolata* (Fig. 1a and b), but not for the alien species (Fig. 1c; significant Number * Origin interaction in Table S1). Subsequent hierarchical diversity-interaction modeling for total biomass of the native species and *P. lanceolata* showed that the model including separate-pairwise GCF interactions had the best fit (Table S2). This indicates that the negative effect of GCF number was due to identities of the GCFs as well as their specific pairwise interactions.

### Responses of plant biomass and PSFs to multiple GCFs in the testing phase

In the testing phase, plant biomass was significantly influenced by the number of GCFs and the soil-conditioning treatment, but there was no main effect of species origin (Table S3). However, all of the interaction terms among those three factors were significant (Table S3), indicating that the abovementioned effects were context-dependent. Specifically, irrespective of species origin, increasing the number of GCFs did not alter plant biomass when grown on conspecific soil (Fig. S3a and b), but significantly decreased plant biomass when grown on unplanted soil and soil trained by *P. lanceolata* (Fig. S3c–f). Furthermore, these negative effects of GCF number were stronger in native than in alien species (Fig. S3c–f; significant Number * Origin interaction in Table S3). Most of the single GCFs, however, did not alter plant biomass regardless of species origin and soil-conditioning treatment (Fig. S4). The hierarchical diversity-interaction modeling for total biomass of both native and alien species grown on unplanted soil and soil trained by *P. lanceolata* showed that the model including separate-pairwise GCF interactions had the best fit (Table S4). This indicates that the negative effect of GCF number resulted from identities of the GCFs and their specific pairwise interactions. Similar to plant total biomass, we also found that plant root mass fraction decreased with an increasing number of GCFs when grown on unplanted soil and soil trained by *P. lanceolate*, but remained unchanged when grown on conspecific soil (Fig. S5).

**Figure 2.**
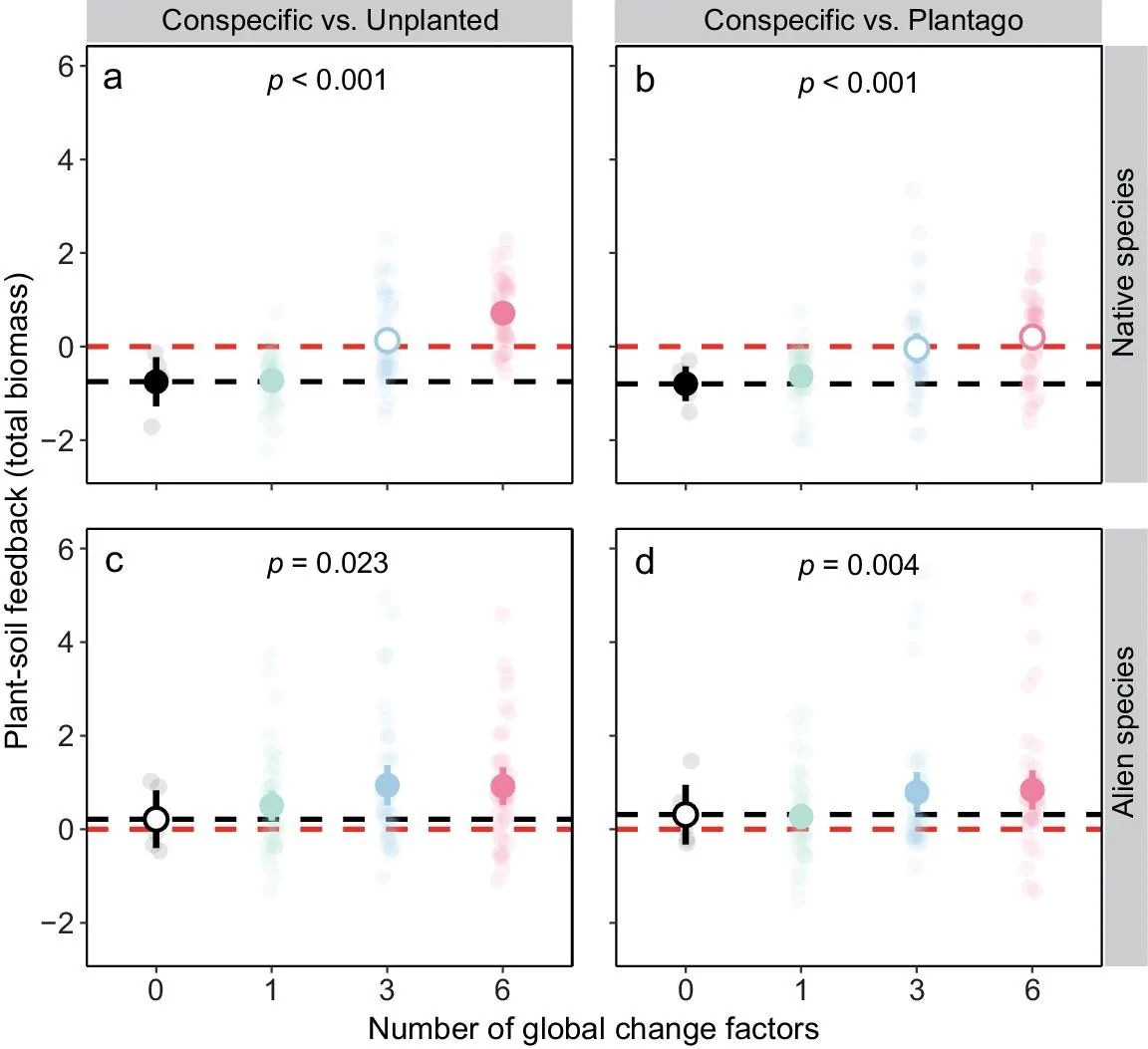
PSFs of native and alien species under different numbers of GCFs. Total PSFs were calculated by comparing total biomass of plants grown on conspecific soil with those on unplanted soil (a, c), and specific PSFs were calculated by comparing total biomass of plants on conspecific soil with those on soil trained by the heterospecific control species *P. lanceolata* (b, d). Points with error bars are means and 95% confidence intervals. Filled points indicate PSFs significantly different from zero, and open points indicate non-significant PSFs. The black dashed line indicates the mean of PSFs under the ambient condition without GCFs, and the red dashed line indicates zero.

To quantify PSFs on total biomass, we calculated log-response ratios of the biomass on conspecific soil versus the biomass on unplanted soil (i.e. total PSFs) and versus the biomass on soil trained by *P. lanceolata* (i.e. specific PSFs). We found that the number of GCFs, species origin and PSF type all had significant effects on PSFs (Table 1). In particular, both total and specific PSFs were positively related to the number of GCFs, and these relationships were stronger in native than in alien species (Fig. 2; significant Number * Origin interaction in Table 1). For native species, total and specific PSFs were negative in treatments without GCFs or with single GCFs, but shifted to neutral or positive in 3- and 6-GCF treatments (Fig. 2a and b). However, for alien species, total and specific PSFs were neutral in treatments without GCFs and became more positive as the number of GCFs increased (Fig. 2c and d). Although there were negative, neutral and positive specific and total PSF effect sizes for the single GCFs, none of these PSF effects was consistent across species origin and PSF type (Fig. S6). The hierarchical diversity-interaction modeling for total PSFs of native species showed that the model including separate-pairwise GCF interactions had the best fit (Table S5). However, for specific PSFs of native species as well as total and specific PSFs of alien species, adding GCF identities or GCF interactions did not significantly improve the model (Table S5).

**Table 1.** linear mixed-effects model (LMM) testing of the effects of GCF number, species origin, PSF type and their interactions on PSFs.

Fixed effects	Degrees of freedom	χ^2^	*P*
GCF number	1	36.41	**<0.001**
Species origin	1	7.27	**0.007**
PSF type	1	4.85	**0.028**
Number * Origin	1	9.66	**0.002**
Number * Type	1	1.76	0.185
Origin * Type	1	0.08	0.783
Number * Origin * Type	1	4.47	**0.035**

Random effects	Standard deviation
GCF treatment	0.097
Family	0.092
Family/species	0.468
Cage ID	0.124
Family * GCF treatment	0.387
Residual	0.835

### Factors driving PSF responses to multiple GCFs

For native species, increasing the number of GCFs significantly increased the colonization by AMF when grown on conspecific soil, but significantly decreased AMF colonization on unplanted soil and soil trained by *P. lanceolata* (Fig. S7a–c and Table S6). However, for alien species, the number of GCFs had no significant effect on AMF colonization irrespective of soil-conditioning treatment (Fig. S7d–f and Table S6).

**Figure 3.**
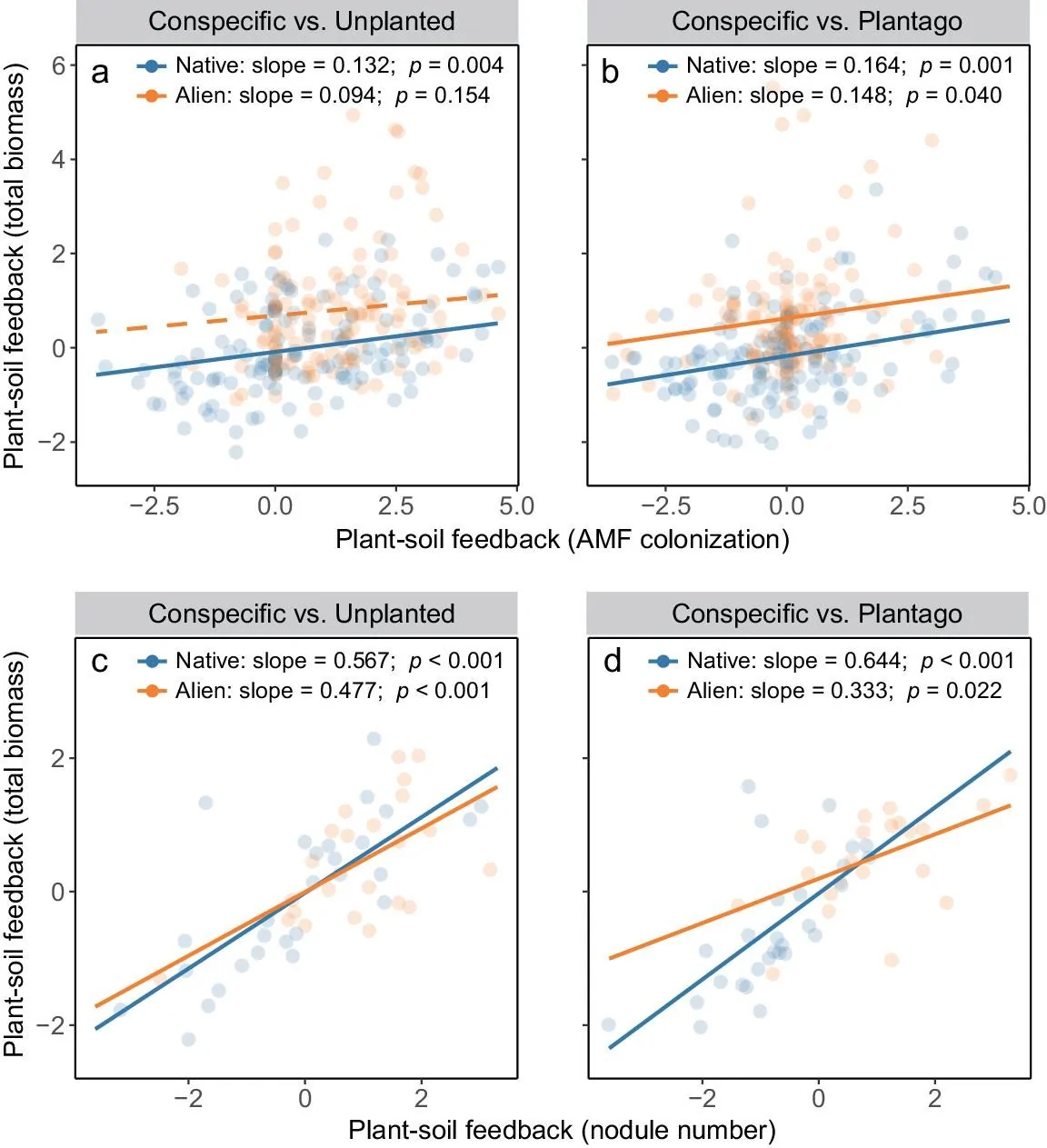
Relationships of PSFs based on total biomass with PSFs based on the colonization by AMF of the 10 species and PSFs based on root nodule number of the two legume species. Total PSFs were calculated by comparing total biomass, AMF colonization or nodule number of plants grown on conspecific soil with those on unplanted soil (a, c), and specific PSFs were calculated by comparing total biomass, AMF colonization or nodule number of plants on conspecific soil with those on soil trained by the heterospecific control species *P. lanceolata* (b, d). In panels (a) and (b), the sample size is 130 (native) and 128 (alien) in conspecific/unplanted comparisons, 134 (native) and 127 (alien) in conspecific/*Plantago* comparisons. In panels (c) and (d), the sample size is 28 (native) and 23 (alien) in conspecific/unplanted comparisons, 28 (native) and 22 (alien) in conspecific/*Plantago* comparisons.

To quantify PSF effects on AMF colonization, we calculated log-response ratios of the AMF colonization of plants on conspecific soil versus the colonization on unplanted soil (i.e. total PSFs) or soil trained by *P. lanceolata* (i.e. specific PSFs). We found that AMF-PSFs were positively related to the number of GCFs in native species, but not in alien species (Fig. S8 and Table S7). Regardless of PSF type, biomass-PSFs were positively associated with AMF-PSFs in native species (Fig. 3a and b and Table S8). However, for alien species, there was only a positive relationship between specific biomass-PSFs and specific AMF-PSFs (Fig. 3b).

Furthermore, increasing the number of GCFs significantly decreased nodule number of the native legume species (*Lotus corniculatus*) when grown on unplanted soil and soil trained by *P. lanceolata*, but not on its conspecific soil, and GCF number had no significant effect on nodule number of the alien legume species (*Lupinus polyphyllus*) in all three soil-conditioning treatments (Fig. S9 and Table S9). These patterns translated into positive effects of GCF number on nodule-PSFs in the native legume species, but not in the alien legume species (Fig. S10 and Table S10). However, irrespective of PSF type and species origin, biomass-PSFs were positively associated with nodule-PSFs (Fig. 3c and d and Table S11).

**Figure 4.**
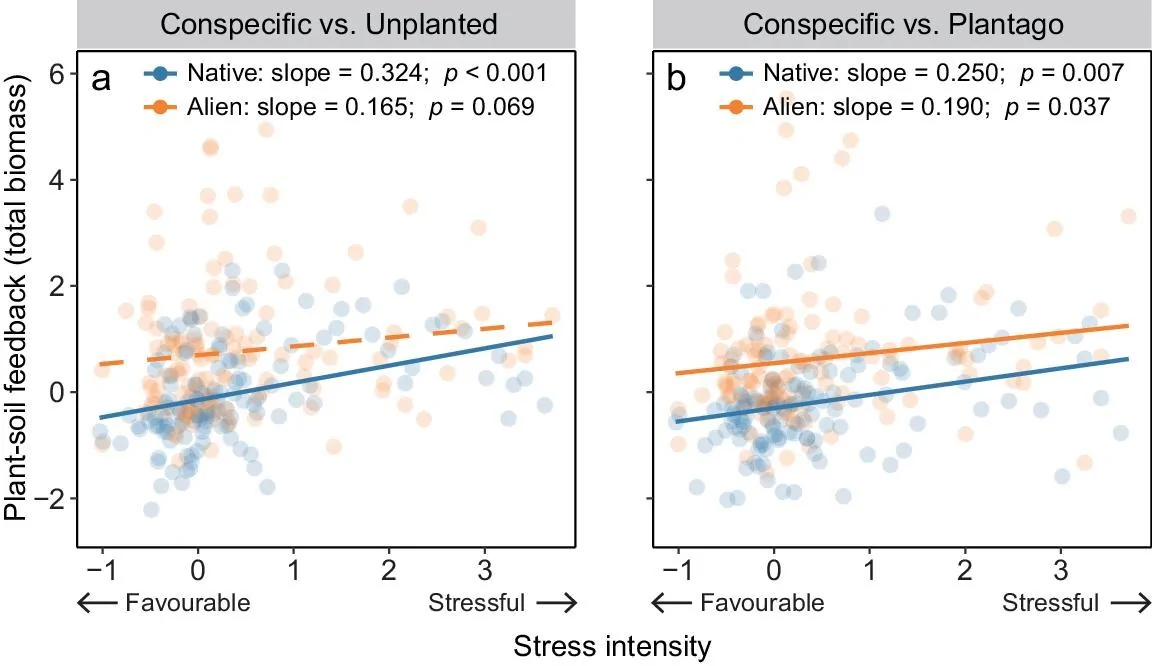
Relationships between PSFs and stress intensity. Total PSFs were calculated by comparing total biomass of plants grown on conspecific soil with those on unplanted soil (a), and specific PSFs were calculated by comparing total biomass of plants on conspecific soil with those on soil trained by the heterospecific control species *P. lanceolata* (b). Stress intensity was calculated by comparing total biomass of *P. lanceolata* in the training phase between treatments without and with GCFs. Positive values of stress intensity indicate that GCFs suppress plant growth and thus provide a stressful condition, and negative values indicate that GCFs promote plant growth and thus provide a favorable condition. The sample size is 130 (native) and 128 (alien) in conspecific/unplanted comparisons, 134 (native) and 128 (alien) in conspecific/*Plantago* comparisons.

As different GCF treatments had negative or positive effects on plants, resulting in either stressful or favorable environmental conditions, we used total biomass of the control species *P. lanceolata* in the training phase to quantify stress intensity. We found that environments became more stressful as the number of GCFs increased (Fig. S11). Across PSF types and species origins, biomass-PSFs were positively associated with stress intensity (Fig. 4 and Table S12), indicating that PSFs became more positive under stressful environmental conditions.

## DISCUSSION

While the individual impacts of some single GCFs on PSFs have been well documented [20,22,27,38], it remains poorly understood how PSFs respond to multiple coacting GCFs and whether such responses differ between alien and native plant species. Here, our PSF experiment encompassing multiple herbaceous plant species and diverse GCF treatments demonstrated that increasing the number of GCFs caused a directional change in PSFs. In particular, PSFs of native species shifted from negative to neutral or positive with an increasing number of GCFs, suggesting that multiple coacting GCFs may destabilize species coexistence and even lead to species loss in native plant communities. This negative consequence may further be exacerbated by non-native plant invasions because multiple coacting GCFs not only enhanced the positive PSFs of alien species but also inhibited the growth of native species more strongly than aliens. Taken together, these findings suggest that multiple coacting GCFs may drive grassland communities toward a dominance of alien species though changes in PSFs.

Under the ambient condition without GCF manipulations, we found that total and specific PSFs were negative in native species, suggesting that the accumulation of both specialist and generalist soil pathogens may contribute to reduced growth of native species [17,39]. Supporting this pathogen-driven mechanism, we found that the use of fungicide, which may have killed many fungal pathogens, shifted PSFs from negative to neutral (Fig. S6a and b). Indeed, this finding is consistent with previous findings that fungicide application alleviates negative PSFs experienced by native plant species [38]. Furthermore, we observed neutral PSFs in the insecticide treatment (Fig. S6a and b), possibly because release from insect herbivores allowed plants to invest more in the production of chemical defenses against other antagonists [40].

Instead, alien species experienced neutral PSFs under the ambient condition, possibly as a result of release from specialist pathogens that would attack them in their native ranges [41,42]. Given that alien species could still be attacked by generalist pathogens and/or root-feeding nematodes, the neutral PSFs in alien species are likely due to compensating effects of their stronger interactions with soil mutualists in their new ranges [41,42]. This is supported by our result that under the ambient condition, the mean colonization of AMF was much higher in alien species (33.2%) than in natives (9.8%) when grown on conspecific soil (Fig. S7a and d). The distinct PSFs between native and alien plant species may also arise from intrinsic divergence in functional traits [43,44]. In particular, invasive alien plants often exhibit a fast-growth strategy [45], characterized by higher specific leaf area, specific root length and root density—functional traits linked to more positive PSFs [46–48]. Overall, our results align with previous findings that native species often experience stronger negative PSFs than aliens under ambient conditions [33–36], suggesting that PSFs may act as an important driver of plant invasions.

As hypothesized, we found that negative PSFs in native species weakened and even became positive with an increasing number of GCFs, particularly when the GCFs resulted in increasing stress intensity, possibly because the stressful environmental conditions suppressed soil pathogens that prefer more benign conditions [25,29]. Although our study could not provide direct molecular evidence, we reanalyzed the fungal sequence data from another experiment with a similar GCF pool [7] and found that the relative abundance of plant pathogens decreased significantly with increasing GCF number (Fig. S12). On the other hand, the positive response of PSFs in native species may also result from the enhanced role of soil mutualists under stressful environmental conditions [25]. This is supported by our findings that biomass-PSFs were positively associated with AMF-PSFs across the five native species and with nodule-PSFs in the native legume species. Nevertheless, we acknowledge that the inclusion of fungicide in our GCF pool may confound the mechanistic interpretation of AMF effects due to the potential non-target effects of fungicides on AMF [49,50]. We therefore conducted subset analyses by excluding treatments involving fungicides and found that the patterns remained unchanged (Figs S13 and S14, Tables S13 and S14), consistent with minimal fungicide effects on AMF colonization in our experiment (Fig. S15 and Table S15).

PSFs in alien species became more positive as the number of GCFs increased, but the role of AMF was only evident for specific PSFs, suggesting that the positive response of PSFs in alien species may also be related to other trophic or functional groups in soil microbial communities (e.g. saprotrophs). For example, Xue *et al.* [30] found that as the number of GCFs increased, Ascomycota, which play a crucial role in nutrient cycling, became more dominant in the soil fungal community left by the alien plant *H. verticillata*, thereby promoting more positive PSFs. However, the weaker relationship between biomass-PSFs and AMF-PSFs in alien species could also be due to the large variation in AMF utilization among alien species (Fig. S7d–f).

We found that the growth of both native and alien plant species was significantly inhibited by an increasing GCF number in control soils. However, this GCF-number effect disappeared in conspecific soil, which directly resulted in positive associations between PSFs and the number of GCFs. For native species, the absence of a GCF-number effect in conspecific soil is probably due to the combined effects of high pathogen pressure in the ambient/single-factor treatments and enhanced mutualist benefits in multiple-factor treatments (Fig. S7a). Instead, given the similar performance of alien species between conspecific and control soils under the ambient condition, the absence of a GCF-number effect probably reflects that mutualists buffered alien species against multiple GCFs in conspecific soil. These results highlight the critical role of a shared history between plants and soil mutualists in mitigating the adverse effects of environmental stress [24]. We further found similar patterns for plant root mass fraction, which decreased with increasing GCF number in control soils. This indicates a shift in resource allocation toward photosynthetic organs, which could enhance carbon acquisition and improve fitness under multifactorial stress.

None of the single GCFs appeared to have a significant effect on PSFs, suggesting that the observed positive response of PSFs to multiple coacting GCFs was unlikely to have emerged from selection effects alone. This is further supported by our hierarchical diversity-interaction modeling analyses on PSFs, which showed that adding GCF identities did not significantly improve the null model, irrespective of species origin and PSF type (Table S5). However, for total PSFs of native species, specific interactions between coacting GCFs contributed significantly to the positive effect of GCF number, highlighting that the consequence of global change at a high-dimensional factor level on PSFs of native species is difficult to predict based on the effects of single GCFs alone. Considering that increasing the number of GCFs influences PSFs via altering the legacies that plants leave in the soil and the performance of the test species [30,51], the unpredictable consequence of multiple GCFs for native PSFs is likely because GCFs interacted with each other in influencing the ability of conditioning plants to produce soil legacies (Table S2) and the performance of the test species (Table S4). For alien species, although specific GCF interactions contributed significantly to the GCF-number effects on their performance in the testing phase (Table S4), this was not the case for their PSFs (Table S5). This may be due to the non-significant effect of GCF number on alien species (and their soil legacies) in the training phase, weakening the contribution of specific GCF interactions to the GCF-number effect on PSFs of alien species.

Our study has several limitations that should be considered. For example, due to logistic constraints, we used only one native species, *P. lanceolata*, as the baseline for measuring specific PSFs instead of pairwise combinations of the 10 other species. This prevents us from predicting possible coexistence among the 10 species in the face of multiple coacting GCFs. In addition, while the duration of our pot experiment is well within the range used in other PSF experiments [22,39], whether the findings obtained here are applicable to field settings at different temporal and spatial scales remains unclear and needs to be tested. Finally, other prevalent factors (e.g. warming) were not included, which should receive particular attention in future research. Despite these caveats, our study provides significant insights into the context-dependence of PSFs between native and alien species under multiple coacting GCFs. Considering that native species were suppressed more strongly than aliens by multiple GCFs, and that alien species still experienced stronger positive PSFs (particularly specific PSFs) compared to natives when facing multiple GCFs, our study highlights that more attention should be paid to controlling plant invasions in a rapidly changing world where multiple GCFs act simultaneously.

## METHODS

### Study species and GCF treatments

To test our hypotheses, we used five pairs of phylogenetically related alien and native herbaceous species in Germany as the study species (Table S16), and selected nine GCFs common to European grasslands. These factors included drought, nitrogen deposition, light pollution, fungicide use, insecticide use, antibiotics, soil salinization, EPDM microplastics and car-tire microplastics (Fig. S1b). Based on these nine GCFs, we created 28 different GCF treatments across the four levels (0, 1, 3 and 6) of increasing GCF numbers (Fig. S1b). Further information on study species and GCF treatments is provided in the Supplementary data.

### PSF experiment

In the training phase, we conditioned soil with each of the aforementioned five native and five alien species to produce conspecific soil. We also produced two types of control soil for each species (Fig. S1a): soil left unplanted and soil conditioned by *P. lanceolata*. In total, we established 840 pots: 10 plant species × 3 soil-conditioning treatments × 28 GCF treatments. Ten weeks after planting seedlings, we harvested plant materials and the soils, and then started the testing phase immediately. Eight weeks after planting, we harvested and dried the plant materials. To explore the possible PSF mechanisms, we randomly collected a small proportion of fine roots for each plant to determine the colonization by AMF, and, for the two legume species, we also counted the number of nodules. Details on the procedure of PSF experiment, the measurement of AMF colonization and the calculation of effect sizes of PSFs and stress intensity are provided in the Supplementary data.

### Statistical analyses

We used linear mixed-effects models (LMMs) to test (i) the effect of GCF number on total biomass of the control species *P. lanceolata* in the training phase, (ii) the interaction effects of GCF number and species origin on total biomass of the 10 species in the training phase, (iii) the interaction effects of GCF number, species origin and soil-conditioning treatment on total biomass, root mass fraction or AMF colonization of the 10 species in the testing phase and (iv) the interaction effects of GCF number, species origin and PSF type on PSFs of the 10 species.

To assess the relationships of PSFs with soil mutualists and stress intensity, we used LMMs to test (i) the interaction effects of PSFs based on AMF colonization (AMF-PSFs), species origin and PSF type on biomass-PSFs of the 10 species, (ii) the interaction effects of nodule-PSFs, species origin and PSF type on biomass-PSFs of the two legume species and (iii) the interaction effects of stress intensity, species origin and PSF type on biomass-PSFs of the 10 species.

To evaluate the contributions of GCF identities and GCF interactions to the significant effect of GCF number, we used the framework of hierarchical diversity-interaction modeling [9,30,52]. Specifically, we ran five hierarchical models specifying different assumptions about the potential contributions of individual GCFs and their interactions to the significant effect of GCF number, and then compared them using likelihood ratio tests (Fig. S16 and Table S17). Detailed model specifications are provided in the Supplementary data. All statistical analyses were conducted in R version 4.1.3 [53].

## Supplementary Material

nwag415_Supplemental_File

## Data Availability

Data and code are archived in Figshare at https://doi.org/10.6084/m9.figshare.28458545.
