# Supplementary material for: Plant–soil feedbacks benefit plant invaders under multiple global change factors
Source: Natl Sci Rev. 2026 Jul 8;13(16):nwag415. doi: 10.1093/nsr/nwag415 (PMC13520119; doi:10.1093/nsr/nwag415)
Supplement: Supplementary file 1 [file nwag415_supplemental_file.docx]

**Supplementary materials**

**Plant-soil feedbacks benefit plant invaders under multiple global change factors**

Cai Cheng^1,2^, Yanjie Liu^3^, Jihua Wu^1,4 *^, Mark van Kleunen^2,5,6^

^1^State Key Laboratory of Wetland Conservation and Restoration, National Observations and Research Station for Wetland Ecosystems of the Yangtze Estuary, Ministry of Education Key Laboratory for Biodiversity Science and Ecological Engineering and Institute of Eco-Chongming, School of Life Sciences, Fudan University, Shanghai 200438, China

^2^Department of Biology, University of Konstanz, Konstanz 78464, Germany

^3^State Key Laboratory of Black Soils Conservation and Utilization, Northeast Institute of Geography and Agroecology, Chinese Academy of Sciences, Changchun 130102, China

^4^State Key Laboratory of Herbage Improvement and Grassland Agro-Ecosystems, College of Ecology, Lanzhou University, Lanzhou 730000, China

^5^Zhejiang Provincial Key Laboratory of Plant Evolutionary Ecology and Conservation, Taizhou University, Taizhou 318000, China

^6^Zhejiang Key Laboratory for Restoration of Damaged Coastal Ecosystems, School of Life Sciences, Taizhou University, Taizhou, Zhejiang, 318000, China

***Correspondence**: Jihua Wu, E-mail: [wjh@lzu.edu.cn](mailto:wjh@lzu.edu.cn)

**Supplementary Methods**

**Study species**

To increase the generalizability of our findings, we tested our hypotheses using five pairs of phylogenetically related alien and native herbaceous species (*Solidago canadensis* L. vs. *Achillea millefolium* L.; *Oenothera biennis* L. vs. *Epilobium hirsutum* L.; *Lupinus polyphyllus* Lindl. vs. *Lotus corniculatus* L.; *Lolium multiflorum* Lam. vs. *Lolium perenne* L.; *Bidens frondosa* L. vs. *Bidens tripartita* L.) from four families (Table S16). Within each pair, the first one is a naturalized alien species in Germany and the latter is a widely distributed native species. To distinguish between specific PSFs and total PSFs, we also included a common native species, *Plantago lanceolata* L., as a heterospecific control for conspecific PSF effects. Seeds were from the seed collection of the botanical garden of the University of Konstanz or ordered from Rieger-Hofmann GmbH, Germany. All seeds were germinated on potting soil in a greenhouse of the botanical garden of the University of Konstanz.

**GCF treatments**

To test the responses of PSFs to multiple GCFs, we selected nine GCFs common to European grasslands, including drought, nitrogen deposition, light pollution, fungicide use, insecticide use, antibiotics, soil salinization, EPDM microplastics and car-tire microplastics (Fig. S1b). These GCFs were chosen because they have different physical, chemical and biological attributes and are likely to continue to increase in magnitude or extent in the near future, and because they could be implemented with the available experimental infrastructure.

(1) Drought: As an important component of global climate change, drought events have increased substantially in frequency and intensity over the last decades in most parts of Europe [1]. Based on the average monthly precipitation (~125 mm) in the growing season (from May to October) between 2013 and 2023 in Konstanz, Germany (http://www.worldweatheronline.com; accessed 20 March 2024), and the top area of the pot, we watered pots without drought with 180 mL every 2 days and drought-treated pots with 180 mL every 4 days. This resulted in an average soil water content of ~39% in pots without drought and ~16% in drought-treated pots. We installed transparent plastic films on the top of the cage to exclude rainfall.
(2) Nitrogen deposition: Nitrogen deposition caused by human activities is an important driver of global change, with an intensity of ~100 kg N ha^-1^ yr^-1^ in most parts of Europe [2,3]. Therefore, to each pot in this treatment, we added 0.5 g of a slow-release nitrogen fertilizer (Floranid N31, Compo Expert, Germany) based on the top area of the pot. We only added nitrogen once during the experimental period because the nitrogen amount (i.e., 0.5 g) is based on a one-year period and Floranid N31 is a slow-release nitrogen fertilizer that is released over a four-month period.
(3) Light pollution: Artificial light at night is an important GCF that disrupts natural circadian rhythms and has profound effects on plant and animal physiology as well as on biotic interactions [4]. For the light-pollution treatment, we used LED spotlights (LED-Strahler Flare 10W, IP 65, 900lm, cool white 6500K; REV Ritter GmbH, Germany), which were installed at the centre of the top of the cage and provided a nighttime light intensity of ~24.5 lx at ground level [2]. The LED spotlights were controlled by a light-sensitive switch that automatically switches on when the natural light is below 50 lx and off when the natural light is above 50 lx.
(4) Fungicide use: For the fungicide treatment, we chose Landor® CT (Syngenta Agro GmbH, Germany), a broad-spectrum fungicide containing three azoles as active agents, because it is widely used in agriculture and horticulture for effective control of a wide range of fungal pathogens [2]. To each pot in this treatment, we added 0.31 μl fungicide, which corresponds to 60% of the maximum amount that should be used per hectare of cropland according to the manufacturer’s directions.
(5) Insecticide use: For the insecticide treatment, we chose Mospilan SG (20% Acetamiprid, Cheminova Deutschland GmbH & Co. KG, Germany), a broad-spectrum, systemic insecticide containing Acetamiprid as the main ingredient, because it is used to control a wide range of insect pests in agriculture. To each pot in this treatment, we added 0.36 mg insecticide according to the manufacturer’s directions.
(6) Antibiotics: Amoxicillin is a commonly used broad-spectrum antibiotic that enters the environment through medical and household use, pharmaceutical industry releases and agricultural farming, with serious impacts on ecosystems and human health [5]. According to the database of Pharmaceuticals in the Environment [6], the mean concentration of amoxicillin in the soil is ~50 mg kg^-1^, which is also within the range of concentrations used in previous studies [5,7,8]. Therefore, we added 60 mg amoxicillin (Zhuhai United Laboratories (Zhongshan) Co., Ltd., China) to each antibiotic-treated pot.
(7) Soil salinization: As an important form of land degradation, soil salinization is often considered a major GCF induced by human activities, such as irrigation during drought periods and road salinization in winter [9]. Soil salinity is usually measured as electrical conductivity, with a conductivity of 4 dS m^-1^ considered to be moderately saline [3]. For the soil-salinization treatment, we added dissolved NaCl to the soil to achieve a salinity of 4 dS m^-1^. To maintain a more or less constant salinity level, electrical conductivity was measured every two weeks and adjusted when required.
(8) EPDM microplastics: The synthetic rubber ethylene propylene diene monomer (EPDM) is widely used in a variety of products (e.g., window wipers) and as infill granules of artificial sport turfs. It is an important source of environmental microplastic pollution [10,11]. To each pot in this treatment, we added 12 g EPDM granules (0.5–2.5 mm diameter, Gummi Appel GmbH & Co. KG, Germany) according to a concentration of 1% (w/w) [2].
(9) Car-tire microplastics: As an important source of terrestrial microplastic pollution, car-tire particles contain polycyclic aromatic hydrocarbons and a variety of heavy metals, with serious impacts on ecosystems [12]. Based on a concentration of 5% (w/w) [13], we added 60 g car-tire particles (1–3 mm diameter, TrendFloor, Germany) to each pot.

Inspired by the experimental design for biodiversity-ecosystem function relationships, following Rillig et al. [14], we created four levels (0, 1, 3 and 6) of increasing GCF numbers based on random sampling from a pool of aforementioned nine GCFs but with the restriction that each GCF was present in an equal number of combinations for each GCF-number level. The zero level was the control without GCF manipulations, and the 1-GCF level included the nine single GCFs. For the 3- and 6-GCF levels, there were nine different combinations, with each of the nine GCFs included in three of the 3-GCF combinations and in six of the 6-GCF combinations. In total, we had 28 different GCF treatments (combinations) across the four levels of increasing GCF numbers (Fig. S1b). We initiated GCF treatments in the training phase and maintained them in the testing phase of the PSF experiment, as GCFs in nature are likely to persist for multiple years. In the training phase, to facilitate establishment of the seedlings, we only imposed drought, fungicide use, insecticide use, antibiotics and soil salinization two weeks after transplanting. For nitrogen deposition, EPDM microplastics and car-tire microplastics, we mixed solid nitrogen fertilizer, EPDM granules and car-tire granules into the soil before seedling transplantation.

**PSF experiment**

***Training phase*.** We conducted the PSF pot experiment outdoor in the botanical garden of the University of Konstanz. We conditioned soil with each of the aforementioned five native and five alien species to produce conspecific soil. To assess total and specific PSFs, respectively, we produced two types of control soil for each species (Fig. S1a): soil left unplanted and soil conditioned by a common native species, *P. lanceolata*. In total, we established 840 pots (12 cm × 12 cm × 13.2 cm): 10 plant species × 3 soil-conditioning treatments × 28 GCF treatments. Each pot was filled with a homogenized mixture of 40% (v/v) sand, 40% vermiculite and 20% field soil. We collected field soils from multiple locations in a natural grassland (47.69 ºN, 9.19 ºE) near the University of Konstanz. The grassland featured a variety of native plant species, such as *Ranunculus repens*, *Poa pratensis*, *P. lanceolate*, *L. corniculatus*, *A. millefolium*, and *L. perenne*. We placed each pot on a plastic dish to preserve water and to avoid cross-contamination. On 14 May 2024, we transplanted into the pots for conspecific or heterospecific soil two similar-sized seedlings of the corresponding plant species, and thinned them to one seedling after the two-week acclimation period. No plants died during the subsequent experimental period. For treatments without light pollution (18 GCF treatments), we randomly assigned pots from two GCF treatments per species to each of nine metal-wire cages (2 m × 2 m × 2 m). For treatments with light pollution (10 GCF treatments), we also randomly assigned pots from two GCF treatments per species to each of five cages. Two adjacent cages were separated by 1 m. Within each cage, 60 pots (10 plant species × 3 soil-conditioning treatments × 2 randomly selected GCF treatments) were randomly assigned to fixed positions and rerandomized every two weeks. Ten weeks after planting, between 23 and 24 July 2024, we harvested the shoot and roots from each pot separately. We immediately dried the plant materials at 70 ºC for 72 h to constant weight and then weighed them. To harvest the soils (to be used in the subsequent testing phase), we put the soil from each pot in a separate plastic bag, picked out the roots and then returned the soil to its original pot. This was also done for the soil in the pots that had been left unplanted.

***Testing phase*.** We started the testing phase immediately after the training phase. On 25 July 2024, we transplanted two similar-sized seedlings of one of the 10 plant species into each pot. So, for each of the 10 plant species, we had one pot for each combination of the three soil-conditioning treatments and 28 GCF treatments. We replaced seedlings that died within two weeks after being transplanted. For pots in which both seedlings had established after the two-week acclimation period, we thinned them to one. However, plants in 42 pots (particularly plants of *S. canadensis*, *A. millefolium* and *L. polyphyllus* in treatments with salinization) died during the subsequent experimental period and were excluded from the analyses. Pots were placed and rearranged as in the training phase. Eight weeks after planting (19 and 21 September 2024), we harvested and dried the plant materials as in the training phase. We divided dry root biomass by dry total biomass to calculate the root mass fraction, which is an indicator of the proportion of total plant biomass allocated to roots.

**AMF colonization and nodule number**

To explore the possible mechanisms underlying the response of PSFs to multiple co-acting GCFs, we randomly collected a small proportion of fine roots for each plant to determine the colonization by AMF, and, for the two legume species, we also counted the number of nodules, before drying the plant materials. We stained roots using the ink-vinegar method according to Vierheilig et al. [15]. In brief, we rinsed root samples with running tap water and then bleached them in a 10% KOH solution in a water bath at 80 °C. We thoroughly rinsed the transparent roots again with running tap water and then stained them with a 5% ink-vinegar solution (Parker QUINK Black, France) for 5 min at 80 °C. After leaving the roots in the ink-vinegar solution for 10 min at room temperature, we rinsed them with running tap water until run-off water was clear. We stored stained roots in a 50% glycerol solution in the dark for at least 1 day and them examined the colonization by AMF using a compound microscope. For each sample, we selected 20 points along each of five root pieces, counting the presence of arbuscules, vesicles or hyphae, resulting in 100 observations per sample.

**Calculation of PSFs and stress intensity**

For each plant species in each GCF treatment, we calculated the effect size (i.e., log-response ratio) of total and specific PSFs using the following formulas:

$\text{Total PSF = ln(}{\text{X}_{\text{Conspecific}}}/{\text{X}_{\text{Unplanted}}}\text{)}$, (1)

$\text{Specific PSF = ln(}{\text{X}_{\text{Conspecific}}}/{\text{X}_{\text{Plantago}}}\text{)}$, (2)

where *X* is the total biomass (or AMF colonization or nodule number; see below) of plants in the pot; subscripts “*Conspecific*”, “*Unplanted*” and “*Plantago*” refer to conspecific soil, unplanted soil and heterospecific soil trained by *P. lanceolata*, respectively. Positive values of PSFs indicate that a species grows better on conspecific soil compared to the control soil, whereas negative values indicate the opposite. Both types of PSFs are commonly used in PSF experiments, but with different implications. Total PSFs assess plant response to changes in whole soil biota and abiotic properties, while specific PSFs assess plant response to changes in species-specific soil biota and abiotic properties. To test the contribution of soil mutualists to PSF variation, we also calculated PSF effect sizes for AMF colonization of each species and nodule number of the two legume species.

Because the different GCF treatments caused negative or positive impacts on plants, resulting in environmental conditions that are either more stressful or more favourable than the control (0 GCF), we used total biomass of the control species *P. lanceolata* in the training phase to quantify stress intensity using the following formula:

$\text{Stress intensity = ln(}{\text{Y}_{\text{Control}}}/{\text{Y}_{\text{GCF}}}\text{)}$, (3)

where *Y* is the total biomass of *P. lanceolata* in the training phase; subscripts “*Control*” and “*GCF*” refer to treatments without and with GCFs, respectively. Positive values of stress intensity indicate that GCFs suppress plant growth and thus provide a stressful condition, and negative values indicate that GCFs promote plant growth and thus provide a favourable condition. We calculated stress intensity based on *P. lanceolata* biomass from the training phase rather than on biomass of the 10 focal plant species in the testing phase to increase the independence between PSFs and stress intensity. However, we also found similar results when using total biomass of the 10 focal species in the training phase to calculate stress intensity (Table S18).

**Statistical analyses**

We used a linear mixed-effects model (LMM) to test the fixed effect of GCF number on total biomass of the control species *P. lanceolata* in the training phase, with GCF treatment and cage id as random factors. We constructed a global LMM to test the fixed effects of GCF number, species origin and their interaction on total biomass of the 10 species in the training phase, with GCF treatment, species nested within family, cage id and the interaction between family and GCF treatment as random factors. To examine the fixed effects of GCF number, species origin, soil-conditioning treatment and their interactions on total biomass, root mass fraction or AMF colonization of the 10 species in the testing phase, we also constructed a global LMM with the same random factors described above. We used a global LMM with the same random factors to test the fixed effects of GCF number, species origin, PSF type and their interactions on PSFs based on biomass (biomass-PSFs) or AMF colonization (AMF-PSFs) of the 10 species. For the two legume species, we used a generalized LMM with a Poisson error distribution to test the fixed effects of GCF number, species origin, soil-conditioning treatment and their interactions on nodule number, with GCF treatment and cage id as random factors. In addition, we used an LMM to test the fixed effects of GCF number, species origin, PSF type and their interactions on PSFs based on nodule number (nodule-PSFs) of the two legume species, with GCF treatment and cage id as random factors. In order to test the difference in plant biomass or PSFs between treatments without and with GCFs, we replaced GCF number in the above LMMs with GCF treatment, and excluded GCF treatment from the random factors. Finally, we used one-sample *t*-tests to examine whether the effect size of PSFs differed from zero [16].

To assess the contribution of soil mutualists to PSF variation, we used a global LMM to test the fixed effects of AMF-PSFs, species origin, PSF type and their interactions on biomass-PSFs, with GCF treatment, species nested within family, cage id and the interaction between family and GCF treatment as random factors. For the two legume species, we constructed an LMM with GCF treatment and cage id as random factors to examine the fixed effects of nodule-PSFs, species origin, PSF type and their interactions on biomass-PSFs. To analyze the relationship between PSFs and stress intensity, we constructed a global LMM to test the fixed effects of stress intensity, species origin, PSF type and their interactions on biomass-PSFs, with GCF treatment, species nested within family, cage id and the interaction between family and GCF treatment as random factors. We checked all LMMs for normality of the residuals and square root transformed data to improve normality when necessary. We removed specific random factors from the model when their incorporation resulted in singular fit warnings due to low variation.

Similar to the biodiversity effects, a significant GCF-number effect could be driven by a higher probability of including GCFs with a strong effect (i.e., a selection effect) or interactions among the GCFs that may be GCF-specific or general. Therefore, we used the framework of hierarchical diversity-interaction modeling [17] to evaluate the contributions of GCF identities and GCF interactions to the significant effect of GCF number on plant biomass or PSFs [2,18]. To do so, we ran five hierarchical models specifying different assumptions about the potential contributions of individual GCFs and their interactions to the significant effect of GCF number, and then compared them using likelihood ratio tests (Fig. S16; Table S17). We generally included the same random factors as in the global LMM described above but removed specific random factors from the models when their incorporation resulted in singularity warnings.

We conducted all statistical analyses in R version 4.1.3 [19]. For LMMs, we used the “lme4” package [20] and then used the “emmeans” package [21] to obtain the statistical significance for GCF number (or AMF-PSFs, nodule-PSFs, stress intensity) and for the difference between treatments without and with GCFs. We used the ‘stats’ package for one-sample *t*-tests.

**Fig. S1. The design of plant-soil feedback (PSF) experiment, treatments of global change factors (GCFs) and the scene of the outdoor common garden. a,** Each of the 10 plant species was tested on conspecific soil, unplanted soil and soil trained by *Plantago lanceolata* to evaluate total and specific PSFs. **b,** Twenty-eight GCF treatments were created based on a pool of nine GCFs and applied during both the training and testing phases. **c,** The PSF experiment was conducted in 14 cages randomly selected from 20 cages in an outdoor garden of the University of Konstanz.

**Fig. S2. Total biomass of plants in different treatments of global change factors in the training phase. a,** The control species *Plantago lanceolata*, **b,** the native species and **c,** the alien species. Points with error bars are means and 95% confidence intervals. The black dashed line indicates the mean of plant biomass under the ambient condition without global change factors. Asterisks indicate significant differences in plant biomass between the treatments without and with global change factors (*** *p* < 0.001, ** *p* < 0.01, * *p* < 0.05).

**Fig. S3. Total biomass of plants grown in different soil-conditioning treatments under different numbers of global change factors in the testing phase.** **a,c,e,** The native species and **b,d,f,** the alien species. Points with error bars are means and 95% confidence intervals. The black dashed line indicates the mean of plant biomass under the ambient condition without global change factors.

**Fig. S4. Total biomass of plants grown in different soil-conditioning treatments in different treatments of global change factors in the testing phase. a,c,e,** The native species and **b,d,f,** the alien species. Points with error bars are means and 95% confidence intervals. The black dashed line indicates the mean of plant biomass under the ambient condition without global change factors. Asterisks indicate significant differences in plant biomass between the treatments without and with global change factors (*** *p* < 0.001, ** *p* < 0.01, * *p* < 0.05).

**Fig. S5. Root mass fraction of plants grown in different soil-conditioning treatments under different numbers of global change factors in the testing phase.** **a,c,e,** The native species and **b,d,f,** the alien species. Points with error bars are means and 95% confidence intervals. The black dashed line indicates the mean of plant root mass fraction under the ambient condition without global change factors.

**Fig. S6. Plant-soil feedbacks (PSFs) of native and alien species in different treatments of global change factors.** Total PSFs were calculated by comparing total biomass of plants on conspecific soil with those on unplanted soil (**a,c**), and specific PSFs were calculated by comparing total biomass of plants on conspecific soil with those on soil trained by the heterospecific control species *Plantago lanceolata* (**b,d**). Points with error bars are means and 95% confidence intervals. Filled points indicate PSFs significantly different from zero, and open points indicate non-significant PSFs. The black dashed line indicates the mean of PSFs under the ambient condition without global change factors, and the red dashed line indicates zero.

**Fig. S7. The root colonization by arbuscular mycorrhizal fungi (AMF) in different soil-conditioning treatments under different numbers of global change factors in the testing phase.** **a–c,** The native species and **d–f,** the alien species. Points with error bars are means and 95% confidence intervals. The black dashed line indicates the mean of AMF colonization under the ambient condition without global change factors.

**Fig. S8. Plant-soil feedbacks (PSFs) based on arbuscular mycorrhizal fungi (AMF) colonization of native and alien species under different numbers of global change factors.** Total PSFs were calculated by comparing AMF colonization of plants grown on conspecific soil with those on unplanted soil (**a,c**), and specific PSFs were calculated by comparing AMF colonization of plants on conspecific soil with those on soil trained by the heterospecific control species *Plantago lanceolata* (**b,d**). Points with error bars are means and 95% confidence intervals. Filled points indicate PSFs significantly different from zero, and open points indicate non-significant PSFs. The black dashed line indicates the mean of PSFs under the ambient condition without global change factors, and the red dashed line indicates zero.

**Fig. S9. Root nodule number of the two legume species grown in different soil-conditioning treatments under different numbers of global change factors in the testing phase. a,** The native *Lotus corniculatus* and **b,** the alien *Lupinus polyphyllus*. Points with error bars are means and 95% confidence intervals. The black dashed line indicates the mean of nodule number under the ambient condition without global change factors.

**Fig. S10. Plant-soil feedbacks (PSFs) based on root nodule number of the native (*Lotus corniculatus*) and alien (*Lupinus polyphyllus*) legume species under different numbers of global change factors.** Total PSFs were calculated by comparing nodule number of plants grown on conspecific soil with those on unplanted soil (**a,c**), and specific PSFs were calculated by comparing nodule number of plants on conspecific soil with those on soil trained by the heterospecific control species *Plantago lanceolata* (**b,d**). Points with error bars are means and 95% confidence intervals. Filled points indicate PSFs significantly different from zero and open points indicate non-significant PSFs. The black dashed line indicates the mean of PSFs under the ambient condition without global change factors, and the red dashed line indicates zero.

**Fig. S11. Stress intensity under different numbers of global change factors (GCFs).** Stress intensity was calculated by comparing total biomass of the control species *Plantago lanceolata* in the training phase between treatments without and with GCFs. Positive values of stress intensity indicate that GCFs suppress plant growth and thus provide a stressful condition, and negative values indicate that GCFs promote plant growth and thus provide a favourable condition.

**Fig. S12. Relative abundance of plant pathogens in soil fungal communities under different numbers of global change factors.** The results were obtained by reanalyzing fungal sequence data from the experiment of Rillig et al. [14] and assigning fungal guilds according to the ‘primary lifestyle’ field of the FungalTraits database [22]. Mycorrhizas were not considered because few were found in the samples, possibly due to the general fungal ITS2 primers used that can underrepresent mycorrhizal diversity, and due to the absence of plants in the experiment.

**Fig. S13. Plant-soil feedbacks (PSFs) of native and alien species under different numbers of global change factors after excluding treatments with fungicides.** Total PSFs were calculated by comparing total biomass of plants grown on conspecific soil with those on unplanted soil (**a,c**), and specific PSFs were calculated by comparing total biomass of plants on conspecific soil with those on soil trained by the heterospecific control species *Plantago lanceolata* (**b,d**). Points with error bars are means and 95% confidence intervals. Filled points indicate PSFs significantly different from zero, and open points indicate non-significant PSFs. The black dashed line indicates the mean of PSFs under the ambient condition without global change factors, and the red dashed line indicates zero.

**Fig. S14. Relationships between plant-soil feedbacks (PSFs) based on total biomass and PSFs based on the colonization by arbuscular mycorrhizal fungi (AMF) of the 10 species after excluding treatments with fungicides.** Total PSFs were calculated by comparing total biomass or AMF colonization of plants grown on conspecific soil with those on unplanted soil (**a**), and specific PSFs were calculated by comparing total biomass or AMF colonization of plants on conspecific soil with those on soil trained by the heterospecific control species *Plantago lanceolata* (**b**). The sample size is 84 (native) and 82 (alien) in conspecific/unplanted comparisons, 87 (native) and 84 (alien) in conspecific/Plantago comparisons.

**Fig. S15. The root colonization by arbuscular mycorrhizal fungi (AMF) between treatments with and without fungicides in different soil-conditioning treatments in the testing phase.** **a–c,** The native species and **d–f,** the alien species. Points with error bars are means and 95% confidence intervals.

**Fig. S16. The framework of hierarchical diversity-interaction modeling to assess contributions of global change factor (GCF) identities and GCF interactions to the significant effects of GCF number [2].** The null model assumes equivalent contributions of all GCFs and no interactions between them. The subsequent models assume more complex effects of how the individual GCFs and their interactions determine the effects of GCF number. The questions that can be answered by comparing specific models are depicted next to the arrows connecting the two models.

**Table S1. Linear mixed-effects model testing the effects of global change factor (GCF) number, species origin and their interaction on total biomass of the 10 focal species in the training phase.**

| Fixed effects | df | χ^2^ | *p* |
| --- | --- | --- | --- |
| GCF number | 1 | 6.01 | **0.014** |
| Species origin | 1 | 0.001 | 0.978 |
| Number * Origin | 1 | 5.57 | **0.018** |
| Random effects | Standard deviation | | |
| GCF treatment | 0.159 | | |
| Family | 0.127 | | |
| Family/species | 0.201 | | |
| Residual | 0.182 | | |

**Table S2. Contributions of global change factor (GCF) identities and GCF interactions to GCF-number effects on total biomass of *Plantago lanceolata* and the native focal species in the training phase.**

| Species | Model (reference model number) | df | AIC | logLik | χ^2^ | *p* |
| --- | --- | --- | --- | --- | --- | --- |
| *Plantago lanceolata* | 1 Null | - | -99.79 | 52.89 | - | - |
|  | 2 GCF identity (1) | 8 | -194.26 | 108.13 | 110.47 (+) | **< 0.001** |
|  | 3 Separate-pairwise GCF  interactions (4) | 16 | -343.35 | 199.68 | 182.70 (+) | **< 0.001** |
|  | 4 Average GCF interaction (2) | 1 | -192.65 | 108.33 | 0.40 | 0.529 |
|  | 5 Additive GCF-specific  interaction contributions (3) | 8 | -342.55 | 191.28 | 16.80 (-) | **0.032** |
| Native focal species | 1 Null | - | -17.27 | 13.63 | - | - |
|  | 2 GCF identity (1) | 8 | -33.37 | 29.68 | 32.10 (+) | **< 0.001** |
|  | 3 Separate-pairwise GCF  interactions (4) | 16 | -49.43 | 54.72 | 49.78 (+) | **< 0.001** |
|  | 4 Average GCF interaction (2) | 1 | -31.65 | 29.83 | 0.28 | 0.594 |
|  | 5 Additive GCF-specific  interaction contributions (3) | 8 | -57.33 | 50.67 | 8.10 | 0.424 |

Note: The numbers and names of the models correspond to those in Fig. S16. The AIC and log-likelihood (logLik) are given for each model. Four model comparisons based on log-likelihood ratio tests are conducted to answer the four questions shown in Fig. S16. The number of the reference model is shown in brackets after the model of interest. For the model comparisons, the degrees of freedom (df), the χ^2^ test statistic and the *p*-value are provided. The + and – in brackets after the χ^2^ value indicate whether the model is better or worse than its reference model.

**Table S3. Linear mixed-effects model testing the effects of global change factor (GCF) number, species origin, soil-conditioning treatment and their interactions on total biomass of plants in the testing phase.**

| Fixed effects | df | χ^2^ | *p* |
| --- | --- | --- | --- |
| GCF number | 1 | 10.88 | **<0.001** |
| Species origin | 1 | 0.12 | 0.731 |
| Soil-conditioning treatment | 2 | 9.56 | **0.008** |
| Number * Origin | 1 | 17.62 | **<0.001** |
| Number * Soil | 2 | 51.46 | **<0.001** |
| Origin * Soil | 2 | 49.00 | **<0.001** |
| Number * Origin * Soil | 2 | 15.39 | **<0.001** |
| Random effects | Standard deviation | | |
| GCF treatment | 0.110 | | |
| Family | 0.047 | | |
| Family/species | 0.205 | | |
| Cage id | 0.013 | | |
| Family * GCF treatment | 0.068 | | |
| Residual | 0.169 | | |

**Table S4. Contributions of global change factor (GCF) identities and GCF interactions to GCF-number effects on total biomass of native and alien species grown on unplanted soil and soil trained by *Plantago lanceolata* in the testing phase.**

| Species origin | Model (reference model number) | Unplanted soil | | | | |  | Soil trained by Plantago | | | | |
| --- | --- | --- | --- | --- | --- | --- | --- | --- | --- | --- | --- | --- |
|  |  | df | AIC | logLik | χ^2^ | *p* |  | df | AIC | logLik | χ^2^ | *p* |
| Native | 1 Null | - | -36.06 | 24.03 | - | - |  | - | 5.51 | 2.25 | - | - |
|  | 2 GCF identity (1) | 8 | -43.93 | 35.96 | 23.87 (+) | **0.002** |  | 8 | -24.83 | 25.42 | 46.34 (+) | **< 0.001** |
|  | 3 Separate-pairwise GCF  interactions (4) | 16 | -41.13 | 51.56 | 29.99 (+) | **0.018** |  | 16 | -33.28 | 46.64 | 41.70 (+) | **< 0.001** |
|  | 4 Average GCF interaction (2) | 1 | -43.14 | 36.57 | 1.21 | 0.271 |  | 1 | -23.58 | 25.79 | 0.74 | 0.389 |
|  | 5 Additive GCF-specific  interaction contributions (3) | 8 | -42.36 | 44.18 | 14.77 | 0.064 |  | 8 | -40.45 | 42.22 | 8.83 | 0.357 |
| Alien | 1 Null | - | -19.97 | 14.99 | - | - |  | - | -23.73 | 15.86 | - | - |
|  | 2 GCF identity (1) | 8 | -23.61 | 24.81 | 19.65 (+) | **0.012** |  | 8 | -21.11 | 22.56 | 13.38 | 0.099 |
|  | 3 Separate-pairwise GCF  interactions (4) | 16 | -34.08 | 47.04 | 44.22 (+) | **< 0.001** |  | 16 | -36.28 | 47.14 | 48.95 (+) | **< 0.001** |
|  | 4 Average GCF interaction (2) | 1 | -21.86 | 24.93 | 0.24 | 0.623 |  | 1 | -19.33 | 22.67 | 0.22 | 0.639 |
|  | 5 Additive GCF-specific  interaction contributions (3) | 8 | -40.39 | 42.20 | 9.68 | 0.288 |  | 8 | -41.95 | 41.97 | 10.34 | 0.242 |

Note to the table is the same as for Table S2.

**Table S5. Contributions of global change factor (GCF) identities and GCF interactions to GCF-number effects on total (Conspecific vs. Unplanted) and specific (Conspecific vs. Plantago) plant-soil feedbacks of native and alien species.**

| Species origin | Model (reference model number) | Conspecific vs. Unplanted | | | | |  | Conspecific vs. Plantago | | | | |
| --- | --- | --- | --- | --- | --- | --- | --- | --- | --- | --- | --- | --- |
|  |  | df | AIC | logLik | χ^2^ | *p* |  | df | AIC | logLik | χ^2^ | *p* |
| Native | 1 Null | - | 281.84 | -137.92 | - | - |  | - | 339.16 | -165.58 |  |  |
|  | 2 GCF identity (1) | 8 | 290.75 | -134.37 | 7.09 | 0.527 |  | 8 | 350.57 | -163.28 | 4.60 | 0.800 |
|  | 3 Separate-pairwise GCF  interactions (4) | 16 | 293.06 | -118.53 | 27.37 (+) | **0.038** |  | 16 | 357.38 | -149.69 | 24.41 | 0.081 |
|  | 4 Average GCF interaction (2) | 1 | 288.43 | -132.21 | 4.32 (+) | **0.038** |  | 1 | 349.79 | -161.90 | 2.77 | 0.096 |
|  | 5 Additive GCF-specific  interaction contributions (3) | 8 | 289.52 | -124.76 | 12.46 | 0.132 |  | 8 | 351.51 | -154.76 | 10.13 | 0.256 |
| Alien | 1 Null | - | 380.10 | -186.05 | - | - |  | - | 390.65 | -191.33 | - | - |
|  | 2 GCF identity (1) | 8 | 385.05 | -180.52 | 11.06 | 0.199 |  | 8 | 403.76 | -189.88 | 2.89 | 0.941 |
|  | 3 Separate-pairwise GCF  interactions (4) | 16 | 400.56 | -171.28 | 17.12 | 0.378 |  | 16 | 410.19 | -176.09 | 25.99 | 0.054 |
|  | 4 Average GCF interaction (2) | 1 | 385.68 | -179.84 | 1.37 | 0.243 |  | 1 | 404.17 | -189.09 | 1.59 | 0.208 |
|  | 5 Additive GCF-specific  interaction contributions (3) | 8 | 394.30 | -176.15 | 9.74 | 0.284 |  | 8 | 408.12 | -183.06 | 13.93 | 0.084 |

Note to the table is the same as for Table S2.

**Table S6. Linear mixed-effects model testing the effects of global change factor (GCF) number, species origin, soil-conditioning treatment and their interactions on the colonization by arbuscular mycorrhizal fungi in the testing phase.**

| Fixed effects | df | χ^2^ | *p* |
| --- | --- | --- | --- |
| GCF number | 1 | 3.50 | 0.061 |
| Species origin | 1 | 0.16 | 0.685 |
| Soil-conditioning treatment | 2 | 120.52 | **< 0.001** |
| Number * Origin | 1 | 1.63 | 0.202 |
| Number * Soil | 2 | 16.24 | **< 0.001** |
| Origin * Soil | 2 | 12.36 | **0.002** |
| Number * Origin * Soil | 2 | 15.86 | **< 0.001** |
| Random effects | Standard deviation | | |
| GCF treatment | 0.565 | | |
| Family | 0.756 | | |
| Family/species | 1.900 | | |
| Cage id | 0.444 | | |
| Residual | 1.940 | | |

**Table S7. Linear mixed-effects model testing the effects of global change factor (GCF) number, species origin, plant-soil feedback (PSF) type and their interactions on PSFs based on the colonization by arbuscular mycorrhizal fungi.**

| Fixed effects | df | χ^2^ | *p* |
| --- | --- | --- | --- |
| GCF number | 1 | 9.76 | **0.002** |
| Species origin | 1 | 3.32 | 0.068 |
| PSF type | 1 | 70.17 | **< 0.001** |
| Number * Origin | 1 | 26.14 | **< 0.001** |
| Number * Type | 1 | 1.91 | 0.167 |
| Origin * Type | 1 | 4.05 | **0.044** |
| Number * Origin * Type | 1 | 0.28 | 0.594 |
| Random effects | Standard deviation | | |
| GCF treatment | 0.237 | | |
| Family | 0.256 | | |
| Family/species | 0.263 | | |
| Family * GCF treatment | 0.686 | | |
| Residual | 1.188 | | |

**Table S8. Linear mixed-effects model testing the effects of plant-soil feedbacks (PSFs) based on the colonization by arbuscular mycorrhizal fungi (AMF), species origin, PSF type and their interactions on biomass-PSFs.**

| Fixed effects | df | χ^2^ | *p* |
| --- | --- | --- | --- |
| AMF-PSFs | 1 | 21.24 | **< 0.001** |
| Species origin | 1 | 7.21 | **0.007** |
| PSF type | 1 | 0.52 | 0.470 |
| AMF-PSFs * Origin | 1 | 0.22 | 0.640 |
| AMF-PSFs * Type | 1 | 0.57 | 0.451 |
| Origin * Type | 1 | 0.06 | 0.814 |
| AMF-PSFs * Origin * Type | 1 | 0.04 | 0.848 |
| Random effects | Standard deviation | | |
| GCF treatment | 0.304 | | |
| Species | 0.436 | | |
| Family * GCF treatment | 0.386 | | |
| Residual | 0.834 | | |

**Table S9. Generalized linear mixed-effects model testing the effects of global change factor (GCF) number, species origin, soil-conditioning treatment and their interactions on root nodule number of the legume species in the testing phase.**

| Fixed effects | df | χ^2^ | *p* |
| --- | --- | --- | --- |
| GCF number | 1 | 14.20 | **< 0.001** |
| Species origin | 1 | 3300.82 | **< 0.001** |
| Soil-conditioning treatment | 2 | 472.13 | **< 0.001** |
| Number * Origin | 1 | 12.85 | **< 0.001** |
| Number * Soil | 2 | 717.72 | **< 0.001** |
| Origin * Soil | 2 | 185.77 | **< 0.001** |
| Number * Origin * Soil | 2 | 39.22 | **< 0.001** |
| Random effects | Standard deviation | | |
| GCF treatment | 0.598 | | |
| Cage id | 0.230 | | |

**Table S10. Linear mixed-effects model testing the effects of global change factor (GCF) number, species origin, plant-soil feedback (PSF) type and their interactions on PSFs based on root nodule number of the legume species.**

| Fixed effects | df | χ^2^ | *p* |
| --- | --- | --- | --- |
| GCF number | 1 | 5.63 | **0.018** |
| Species origin | 1 | 55.64 | **< 0.001** |
| PSF type | 1 | 4.80 | **0.028** |
| Number * Origin | 1 | 19.11 | **< 0.001** |
| Number * Type | 1 | 0.90 | 0.342 |
| Origin * Type | 1 | 2.54 | 0.111 |
| Number * Origin * Type | 1 | 1.10 | 0.295 |
| Random effects | Standard deviation | | |
| GCF treatment | 0.634 | | |
| Residual | 0.902 | | |

**Table S11. Linear mixed-effects model testing the effects of plant-soil feedbacks (PSFs) based on nodule number, species origin, PSF type and their interactions on biomass-PSFs.**

| Fixed effects | df | χ^2^ | *p* |
| --- | --- | --- | --- |
| Nodule-PSFs | 1 | 67.40 | **< 0.001** |
| Species origin | 1 | 0.16 | 0.691 |
| PSF type | 1 | 0.001 | 0.981 |
| Nodule-PSFs * Origin | 1 | 1.94 | 0.164 |
| Nodule-PSFs * Type | 1 | 0.02 | 0.894 |
| Origin * Type | 1 | 0.20 | 0.656 |
| Nodule-PSFs * Origin * Type | 1 | 0.78 | 0.378 |
| Random effects | Standard deviation | | |
| GCF treatment | 0.156 | | |
| Cage id | 0.215 | | |
| Residual | 0.690 | | |

**Table S12. Linear mixed-effects model testing the effects of stress intensity, species origin, plant-soil feedback (PSF) type and their interactions on biomass-PSFs.**

| Fixed effects | df | χ^2^ | *p* |
| --- | --- | --- | --- |
| Stress intensity | 1 | 14.16 | **<0.001** |
| Species origin | 1 | 7.00 | **0.008** |
| PSF type | 1 | 4.60 | **0.032** |
| Stress * Origin | 1 | 1.82 | 0.177 |
| Stress * Type | 1 | 0.11 | 0.745 |
| Origin * Type | 1 | 0.09 | 0.763 |
| Stress * Origin * Type | 1 | 0.42 | 0.518 |
| Random effects | Standard deviation | | |
| GCF treatment | 0.291 | | |
| Family | 0.102 | | |
| Family/species | 0.465 | | |
| Family * GCF treatment | 0.351 | | |
| Residual | 0.853 | | |

**Table S13. Linear mixed-effects model testing the effects of global change factor (GCF) number, species origin, plant-soil feedback (PSF) type and their interactions on PSFs after excluding treatments with fungicides.**

| Fixed effects | df | χ^2^ | *p* |
| --- | --- | --- | --- |
| GCF number | 1 | 33.25 | **<0.001** |
| Species origin | 1 | 8.59 | **0.003** |
| PSF type | 1 | 3.02 | 0.082 |
| Number * Origin | 1 | 3.11 | 0.078 |
| Number * Type | 1 | 2.78 | 0.095 |
| Origin * Type | 1 | 0.17 | 0.680 |
| Number * Origin * Type | 1 | 3.78 | 0.052 |
| Random effects | Standard deviation | | |
| Species | 0.490 | | |
| Cage id | 0.067 | | |
| Family * GCF treatment | 0.419 | | |
| Residual | 0.764 | | |

**Table S14. Linear mixed-effects model testing the effects of plant-soil feedbacks (PSFs) based on the colonization by arbuscular mycorrhizal fungi (AMF), species origin, PSF type and their interactions on biomass-PSFs after excluding treatments with fungicides.**

| Fixed effects | df | χ^2^ | *p* |
| --- | --- | --- | --- |
| AMF-PSFs | 1 | 24.24 | **< 0.001** |
| Species origin | 1 | 7.82 | **0.005** |
| PSF type | 1 | 0.01 | 0.912 |
| AMF-PSFs * Origin | 1 | 0.12 | 0.724 |
| AMF-PSFs * Type | 1 | 0.48 | 0.488 |
| Origin * Type | 1 | 0.04 | 0.850 |
| AMF-PSFs * Origin * Type | 1 | 0.12 | 0.727 |
| Random effects | Standard deviation | | |
| GCF treatment | 0.305 | | |
| Species | 0.467 | | |
| Family * GCF treatment | 0.404 | | |
| Residual | 0.757 | | |

**Table S15. Linear mixed-effects model testing the effects of fungicide, global change factor (GCF) number, species origin, soil-conditioning treatment and their interactions on the colonization by arbuscular mycorrhizal fungi in the testing phase.**

| Fixed effects | df | χ^2^ | *p* |
| --- | --- | --- | --- |
| Fungicide | 1 | <0.001 | 0.997 |
| GCF number | 1 | 2.35 | 0.126 |
| Species origin | 1 | 0.16 | 0.688 |
| Soil-conditioning treatment | 2 | 121.92 | **< 0.001** |
| Fungicide * Number | 1 | 0.06 | 0.801 |
| Fungicide * Origin | 1 | 0.02 | 0.883 |
| Number * Origin | 1 | 1.05 | 0.306 |
| Fungicide * Soil | 2 | 1.20 | 0.548 |
| Number * Soil | 2 | 11.72 | **0.003** |
| Origin * Soil | 2 | 12.62 | **0.002** |
| Fungicide * Number * Origin | 1 | 0.23 | 0.634 |
| Fungicide * Number * Soil | 2 | 9.00 | **0.011** |
| Fungicide * Origin * Soil | 2 | 1.98 | 0.371 |
| Number * Origin * Soil | 2 | 12.84 | **0.002** |
| Fungicide * Number * Origin * Soil | 2 | 6.21 | **0.045** |
| Random effects | Standard deviation | | |
| GCF treatment | 0.611 | | |
| Family | 0.747 | | |
| Family/species | 1.903 | | |
| Cage id | 0.431 | | |
| Residual | 1.929 | | |

**Table S16. Information on the plant species used in this experiment.**

| Species | Family | Origin | Sowing date (Year 2024) | |  |
| --- | --- | --- | --- | --- | --- |
|  |  |  | Training phase | Testing phase | Seed source |
| *Solidago canadensis* L. | Asteraceae | Alien | 22 April | 24 June | University of Konstanz |
| *Achillea millefolium* L. |  | Native | 22 April | 24 June | Rieger-Hofmann GmbH |
| *Oenothera biennis* L. | Onagraceae | Alien | 22 April | 24 June | Rieger-Hofmann GmbH |
| *Epilobium hirsutum* L. |  | Native | 22 April | 24 June | University of Konstanz |
| *Lupinus polyphyllus* Lindl. | Fabaceae | Alien | 29 April | 01 July | University of Konstanz |
| *Lotus corniculatus* L. |  | Native | 29 April | 01 July | Rieger-Hofmann GmbH |
| *Lolium multiflorum* Lam. | Poaceae | Alien | 29 April | 01 July | University of Konstanz |
| *Lolium perenne* L. |  | Native | 29 April | 01 July | Rieger-Hofmann GmbH |
| *Bidens frondosa* L. | Asteraceae | Alien | 29 April | 01 July | University of Konstanz |
| *Bidens tripartita* L. |  | Native | 29 April | 01 July | University of Konstanz |
| *Plantago lanceolata* L. | Plantaginaceae | Native | 29 April | – | Rieger-Hofmann GmbH |

**Table S17. The five hierarchical models disentangling contributions of global change factor (GCF) identities and GCF interactions to the significant effects of GCF number [2].**

| Number | Formula |
| --- | --- |
| Model 1 (Null model) | Variable ~ initial abundance of GCFs (M) |
| Model 2 | Variable ~ M + GCF identities |
| Model 3 | Variable ~ M + GCF identities + separate-pairwise GCF interactions |
| Model 4 | Variable ~ M + GCF identities + average GCF interaction |
| Model 5 | Variable ~ M + GCF identities + average GCF-specific interactions |

Note: The first model (i.e., the null model) assumes equal contributions of all GCFs and no interactions between them, which only includes the centered sum of the GCFs of each treatment (M) as a fixed effect. M accounts for differences in the “initial abundances” of GCFs, indicating that the other model terms are interpreted based on the average initial abundance, and is also included in the other four models. This way, we could include the GCFs’ relative proportions in each GCF combination, instead of just considering GCF presence, while taking into account that, with increasing GCF number, the relative proportion of each individual GCF is automatically reduced. In the second model, the GCF identities (i.e., the proportion of each GCF in a specific GCF combination) are added, assuming that individual GCFs contribute differently to the GCF-number effect. In the third model, separate-pairwise interactions between GCFs are added, assuming that both GCF identities and specific pairwise interactions between GCFs contribute to the GCF-number effect. In the fourth model, separate-pairwise GCF interactions are replaced by an average interaction effect, assuming that all pairwise GCF interactions contribute equally to the GCF-number effect. In the fifth model, the average interaction effect of the fourth model is replaced by average GCF-specific interaction effects, assuming that the contribution of each GCF to a pairwise interaction is constant.

**Table S18. Linear mixed-effects model testing the effects of stress intensity, species origin, plant-soil feedback (PSF) type and their interactions on biomass-PSFs when using total biomass of the 10 focal species in the training phase to calculate stress intensity.**

| Fixed effects | df | χ^2^ | *p* |
| --- | --- | --- | --- |
| Stress intensity | 1 | 5.58 | **0.018** |
| Species origin | 1 | 8.18 | **0.004** |
| PSF type | 1 | 4.44 | **0.035** |
| Stress * Origin | 1 | 0.49 | 0.486 |
| Stress * Type | 1 | 1.57 | 0.211 |
| Origin * Type | 1 | 0.02 | 0.901 |
| Stress * Origin * Type | 1 | 0.91 | 0.340 |
| Random effects | Standard deviation | | |
| GCF treatment | 0.338 | | |
| Species | 0.458 | | |
| Family * GCF treatment | 0.365 | | |
| Residual | 0.852 | | |

**Supplementary References**

1. Markonis, Y. et al. The rise of compound warm-season droughts in Europe. *Sci. Adv.* **7**, eabb9668 (2021).
2. Speißer, B., Wilschut, R. A. & van Kleunen, M. Number of simultaneously acting global change factors affects composition, diversity and productivity of grassland plant communities. *Nat. Commun.* **13**, 7811 (2022).
3. Yang, G. W. et al. Multiple anthropogenic pressures eliminate the effects of soil microbial diversity on ecosystem functions in experimental microcosms. *Nat. Commun.* **13**, 4260 (2022).
4. Liu, Y. J. & Heinen, R. Plant invasions under artificial light at night. *Trends Ecol. Evol.* **39**, 703-705 (2024).
5. Mao, L. Y. et al. Ecological succession of abundant and rare subcommunities during aerobic composting in the presence of residual amoxicillin. *J. Hazard. Mater.* **465**, 133456 (2024).
6. Graumnitz, S. & Jungmann, D. *Pharmaceuticals in the environment, update for the period 2017-2020*. Germany Environment Agency (2021). https://www.umweltbundesamt.de/publikationen/the-database-pharmaceuticals-in-the-environment-0.
7. Cela-Dablanca, R. et al. Relevance of sorption in bio-reduction of amoxicillin taking place in forest and crop soils. *Environ. Res.* **208**, 112753 (2022).
8. Rodríguez-González, L. et al. Direct toxicity of six antibiotics on soil bacterial communities affected by the addition of bio-adsorbents. *Environ. Pollut.* **322**, 121161 (2023).
9. Daliakopoulos, I. N. et al. The threat of soil salinity: A European scale review. *Sci. Total Environ.* **573**, 727-739 (2016).
10. van Kleunen, M. et al. A microplastic used as infill material in artificial sport turfs reduces plant growth. *Plants People Planet* **2**, 157-166 (2020).
11. Lassen, C. et al. *Microplastics: Occurrence, effects and sources of releases to the environment in Denmark*. Copenhagen, Denmark: Danish Environmental Protection Agency (2015).
12. Baensch-Baltruschat, B. et al. Tyre and road wear particles (TRWP)-A review of generation, properties, emissions, human health risk, ecotoxicity, and fate in the environment. *Sci. Total Environ.* **733**, 137823 (2020).
13. Leifheit, E. F. et al. Tire abrasion particles negatively affect plant growth even at low concentrations and alter soil biogeochemical cycling. *Soil Ecol. Lett.* **4**, 409-415 (2022).
14. Rillig, M. C. et al. The role of multiple global change factors in driving soil functions and microbial biodiversity. *Science* **366**, 886-890 (2019).
15. Vierheilig, H., Coughlan, A. P., Wyss, U. & Piché, Y. Ink and vinegar, a simple staining technique for arbuscular-mycorrhizal fungi. *Appl. Environ. Microbiol.* **64**, 5004-5007 (1998).
16. Brinkman, E. P., Van der Putten, W. H., Bakker, E. J. & Verhoeven, K. J. F. Plant-soil feedback: experimental approaches, statistical analyses and ecological interpretations. *J. Ecol.* **98**, 1063-1073 (2010).
17. Kirwan, L. et al. Diversity-interaction modeling: estimating contributions of species identities and interactions to ecosystem function. *Ecology* **90**, 2032-2038 (2009).
18. Xue, W. et al. Number of global change factors alters plant-soil feedbacks via its effect on soil fungal communities. *Soil Biol. Biochem.* **194**, 109443 (2024).
19. R Core Team. *R: A language and environment for statistical computing*. Vienna: R Foundation for Statistical Computing (2022).
20. Bates, D. et al. *lme4: Linear Mixed-Effects Models using 'Eigen' and S4. R package version 1.1-31* (2022).
21. Lenth, R. V. et al. *emmeans: Estimated Marginal Means, aka Least-Squares Means*. R package version 1.8.4-1 (2023).
22. Põlme, S. et al. FungalTraits: a user-friendly traits database of fungi and fungus-like stramenopiles. *Fungal Divers.* **105**: 1-16 (2020).
